# Generalisability of trials of home blood pressure monitoring; a comparison of two UK primary care trials

**DOI:** 10.1186/1745-6215-12-S1-A99

**Published:** 2011-12-13

**Authors:** Sally M Kerry, Hugh S Markus, Teck K Khong, Pippa Oakeshott

**Affiliations:** 1 Blizard Institute, Barts and The London School of Medicine and Dentistry, Queen Mary University of London, London, E1 2AB, UK; 2 Stroke and Dementia Research Group, St George’s, University of London, London, SW17 0RE, UK; 3 Basic Medical Sciences, St George’s, University of London, London, SW17 0RE, UK; 4 Population Health Sciences and Education, St George’s, University of London, London, SW17 0RE, UK

## Objective

Blood pressure (BP) monitors are widely available and easy for patients to use. Systematic reviews [1-3] show that home monitoring of BP improves BP control but there is significant heterogeneity between studies, and meta regression has only been able to explain part of the heterogeneity, with concomitant interventions being a possible factor.

Most evidence comes from trials of patients who have poorly controlled blood pressure at baseline, although not usually explicitly stated in trial or review title. However many hypertensive patients using home monitors may have BP below the recommended target

Two large recent UK RCTs [4,5] have been carried out with very different inclusion criteria and interventions. The objective is to compare the main findings of these trials and assess how far difference between the trial populations might explain the apparent difference in the efficacy of the intervention between the two trials.

## Methods

In 2007-9, 381 hypertensive stroke patients were recruited from stroke services in London, and randomly allocated, at home, to either home BP monitoring (n=187), with ongoing telephone support from a nurse, or usual care (n=194). Patients were included without any restriction on baseline BP [4].

The TASMINH trial [5] randomised 527 primary care patients with baseline BP between 140/90 and 200/100, to a self management program (n=263) or control (n=264). The program involved two training sessions for patients, so that they could increase their medications when necessary, without consulting the GP.

The primary endpoint for both trials was change in mean systolic BP between baseline and 12 months follow up after adjusting for baseline BP. A post hoc subgroup analysis of those patients in the stroke study with baseline BP between 140/90 and 200/100 was carried out for comparison.

## Results

Follow up rates for survivors were over 90% in both trials.

Intervention effect in the TASMINH trial was 5.4(2.4 to 8.5); n=480, and was 0.3(-3.6 to 4.80) in the stroke trial; n=337.

Of 1650 patients assessed for eligibility in the TASMINH trial, 916 (55%) had BP less than 140/90 and 48 had BP above 200/100. These patients were excluded prior to randomisation.

133 (40%) patients in the stroke trial had baseline BP between 140/90 and 200/100; the intervention effect in this group was 5.9(-0.3 to 12.0) ; p=0.02 for interaction.

## Conclusions

Whether or not patients with controlled hypertension, who comprise more than half the hypertensive population, are included, may explain the difference in effect size between these 2 trials.
